# Ordinary differential equation models of SARS-CoV-2 replication dynamics and antiviral drug efficacies

**DOI:** 10.1038/s44298-026-00183-8

**Published:** 2026-03-13

**Authors:** Timon Kapischke, Simon T. Herrmann, Luca D. Bertzbach, Stephanie Pfaender, Lars Kaderali

**Affiliations:** 1 Institute of Bioinformatics, University Medicine Greifswald, Greifswald, Germany; 2 Research Unit Emerging Viruses, Leibniz Institute of Virology (LIV), Hamburg, Germany; 3 Institute of Virology and Cell Biology, University of Lübeck, Lübeck, Germany

**Keywords:** Computational biology and bioinformatics, Diseases, Mathematics and computing

## Abstract

There is a critical need for precise analysis of virus–host interactions to improve our understanding of infection processes. The integration of quantitative measurements with dynamic mathematical modeling has changed how we perceive cellular infection processes, offering profound insights into how viruses function at the cellular level. Here, we systematically review target cell-limited (TCL) ordinary differential equation (ODE) models related to SARS-CoV-2. We examine the spectrum of available models from basic TCL frameworks to more complex models incorporating antiviral treatments—and highlight key findings, discuss strengths and limitations, and identify a shortage of comprehensive datasets, emphasizing structural identifiability issues.

## Introduction

Coronaviruses are enveloped, positive-sense, single-stranded RNA viruses with among the largest genomes of all RNA viruses^1^. To date, seven coronaviruses are known to infect humans. Of these, four—human coronavirus HCoV-229E, -HKU1, -NL63, and -OC43—are endemic, circulate seasonally worldwide, and typically cause mild, cold-like symptoms^2–4^. Over the past three decades, three pathogenic coronaviruses have emerged. The first was the severe acute respiratory syndrome coronavirus (SARS-CoV) in 2002, followed by the Middle East respiratory syndrome coronavirus (MERS-CoV) in 2012, both of which caused serious outbreaks^5,6^. In late 2019, SARS-CoV-2, the virus responsible for COVID-19, was first identified in Wuhan, China^7^. As the virus rapidly spread globally, leading to unprecedented public health concerns, the World Health Organization (WHO) declared a SARS-CoV-2 pandemic in March 2020. Since then, over 777 million cases of COVID-19 and more than 7 million deaths have been reported to the WHO^8^. Mutations in the viral genome have led to the emergence of several variants of concern (VOCs), characterized by increased transmissibility, immune evasion, and varying degrees of effectiveness of antiviral drugs and vaccines^9^.

A comprehensive understanding of the molecular replication cycle of SARS-CoV-2, including attachment and entry, protein translation, viral replication, and assembly, along with its transmission mechanisms, virus–host interactions (including with the host immune system), and mechanisms of pathogenesis, is essential for developing effective interventions such as antiviral drugs and vaccines.

Mathematical models serve as powerful tools for both understanding the complex mechanisms of viruses and developing effective countermeasures against them^10,11^. These models are invaluable in simulating various aspects of viral behavior, such as the replication of viruses within host cells, the transmission of infection from one cell to another, and the broader dynamics of how infections spread within populations. Additionally, they help to elucidate virus–host interactions, including the processes of pathogenesis and immune responses to infections.

Such models can be used to determine key parameters such as the infection rate and mortality rate of viruses, for example. They can also be used to estimate the basic reproduction number (R_0_) and the duration of an infection. In addition, they can also illustrate the unequal impact of viruses within a heterogeneous population, helping to identify high-risk groups^12^. Beyond analyzing dynamic processes, they allow for the simulation of various scenarios without relying on empirical data. This makes it possible to estimate the potential effects of antiviral intervention strategies, such as how drugs influence viral replication or spread^13,14^. It is important to note that the scale of the model determines its output. For example, a molecular-scale model can represent intracellular viral replication, virus–host interactions like cell-to-cell transmission, or immune responses^15^. On a larger population scale, models can provide insights into organism-to-organism transmission, the identification of risk groups, or patterns of seasonal outbreaks^16–18^.

Over the past years, several mathematical models analyzing the replication of SARS-CoV-2 have been developed and refined. In this review, we focus exclusively on within-host viral dynamics models, ranging from simple target cell-limited (TCL) models to more complex ones that focus on the effect of antiviral drugs, on compartmental structures, and coinfection dynamics.

## Search strategy and selection criteria

We searched the primary databases Web of Science and PubMed, using the keywords “SARS-CoV-2”, “mathematical modeling”, and “cell”. Publications before 2019, as well as those containing the keywords “SEIR”, “SIR”, “PDE”, “DDE”, and “IDE”, were excluded during the search to focus exclusively on studies using TCL/ODE models of SARS-CoV-2, which first emerged in late 2019. We focused on literature published in English between January 1, 2019, and August 10, 2025. In addition to searching the primary databases, Google Scholar was used for a supplementary secondary search with the same search criteria.

**Fig. 1:**
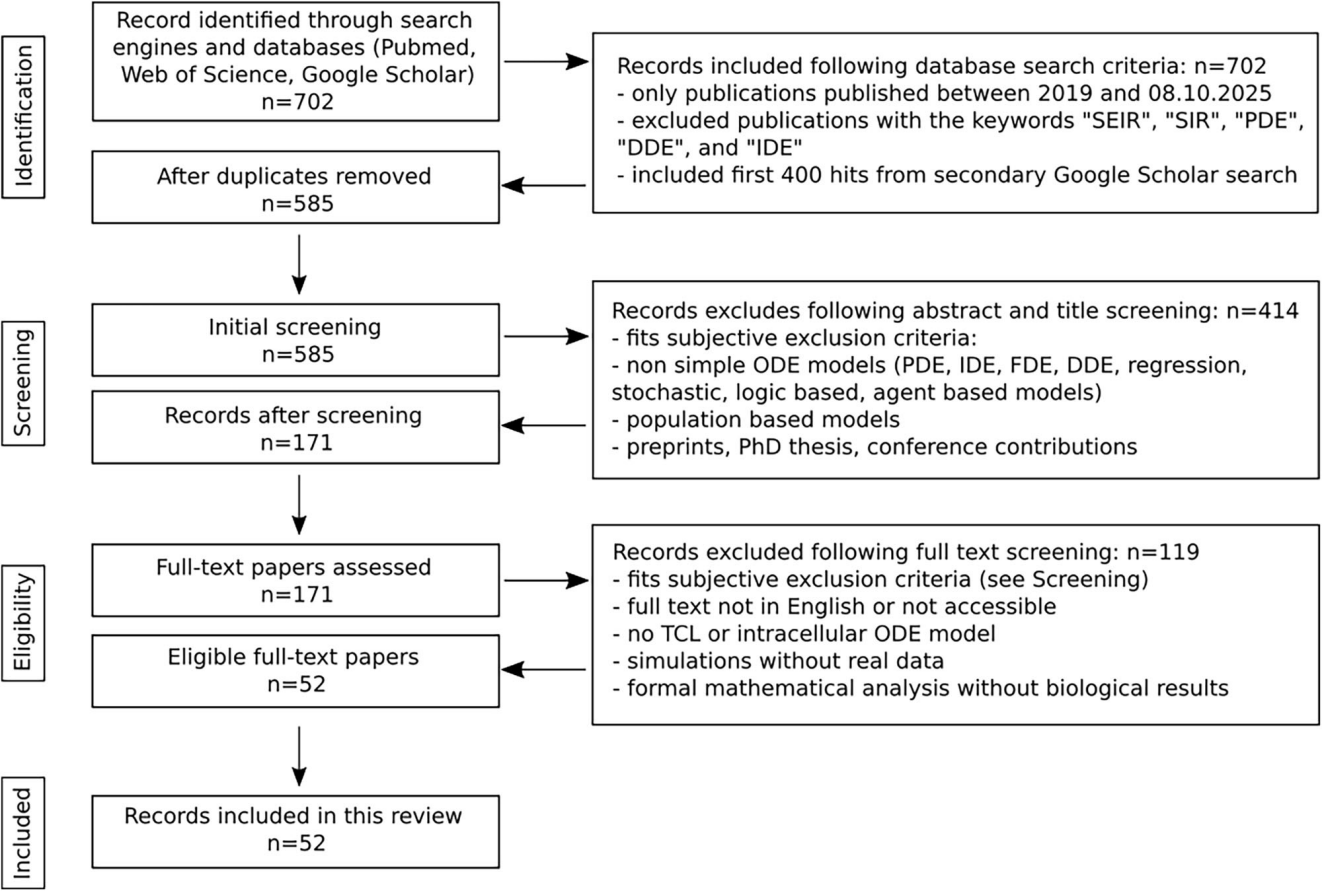
Literature search and study selection workflow. Diagram illustrating the selection strategy used for the literature search. In total, we identified 702 articles from Web of Science, PubMed, and Google Scholar. After applying the indicated exclusion criteria, 52 publications remained (see also Table 1 and Fig. S1).

After removing duplicates, we excluded preprints, theses, review articles, conference papers, epidemiology-based models, and non-simple ordinary differential equation (ODE) models, such as regression, logic-based, stochastic, or agent-based models. Additionally, we excluded models involving partial differential equations (PDEs), integro-differential equations (IDEs), fractional differential equations (FDEs), and delayed differential equations (DDEs). We allowed ODE-based models with delayed activations implemented via Heaviside step function. We excluded studies that did not use real experimental data to inform their models, as well as those that did not focus on TCL models, multiscale models with population dynamics, or mathematical studies that emphasized formal model analysis rather than biological results. For more details, refer to Fig. 1.

In total, we identified 267 articles from Web of Science, 35 from PubMed, and 400 from Google Scholar. After applying our exclusion criteria, 52 publications remained (Table 1).

**Table 1:** Detailed overview of the 52 ODE models reviewed in this study

Reference	Model type	Identifiability analysis	Sensitivity analysis	Data used	Number of equations within the model(s)	Number of unique models	Fitting method	Data used for model fitting	Comments
Sadria et al.^78^	Antiviral	No	Yes	^85,86^	12	1	Not mentioned (MATLAB)	Viral load (VL)	Immune response (IR) parameters adapted from IVA infection
Leander et al.^43^	Antiviral	No	Yes	^87^	14	1	Least-Squares (MATLAB)	VL	IR parameters from literature
Ke et al.^44^	Antiviral	No	Yes	^86,88^	4, 5	2	Nonlinear mixed effects (NLME) (Monolix)	VL	
Wang et al.^26^	Antiviral	No	Yes	^85,89–91^	3, 4, 4	3	Regression modeling strategies (RMS) (R)	VL (human and non-human)	Fixed some IR parameters
Néant et al.^38^	Antiviral	No (but taken into account)	Yes	^38,92^	5, 6	9	Stochastic approximation expectation-maximization (SAEM) algorithm (Monolix 2018R2)	VL	
Zhou et al.^46^	Antiviral	No	Yes	^46,86,89,93–95^	32	4	Python	VL, IR (white blood cells (WBC), neutrophils, lymphocytes, monocytes, cytokines)	
Ghosh^55^	Antiviral	No	Yes	^86^	8	1	fminsearchbnd() (MATLAB)	VL	IR parameters mostly adapted from literature or assumed
Dogra et al.^57^	Antiviral	No	Yes	^96–98^	16	1	Linear least-squares (LLS) (MATLAB R2018a)	VL, IR (CD4^+^ and CD8^+^ T cells, cytokines, antibodies)	Many IR parameters from literature
Yamaguchi et al.^54^	Antiviral	No (but taken into account)	No	^99–101^	6	5	SAEM algorithm (NONMEM v7.4.4)	VL	
Goyal et al.^77^	Antiviral	No	Yes	^86,89,93,94,102,103^	n; > 5	n*	SAEM algorithm (Monolix 2019R2)	VL, pharmacokinetics data (remdesivir, selinexor)	*n precursors cell equations
Al-Darabsah et al.^82^	Antiviral	Yes	Yes	^104,105^	4	1	Differential evolution algorithm (MATLAB); genetic algorithm (R); NMinimize (Wolfram Mathematica)	VL, IR (monocytes, CD4^+^,and CD8^+^ T cells)	
Rana et al.^53^	Antiviral	No	Yes	^106^	8	1	Levenberg–Marquardt algorithm, Nelder-Mead + Random Search, curve_fit() (Python, MATLAB)	VL, IR (cytokines)	Many IR parameters from literature
Zhang et al.^36^	Antiviral	No (but taken into account)	Yes	^107^	5	8	SAEM (R 4.2.1)	VL	
Danchin et al.^51^	Antiviral	No	Yes	^94,108^	5	2	n.a.	VL*, IR** (antibodies)	*1x patient; **Mean from cohort
Reis et al.^47^	Antiviral	No	Yes	^87,109,110^	15	1	Differential Evolution (Python)	VL, IR (antibodies, cytokines)	
Lin et al.^45^	Antiviral	No	Yes	^45^	8	1	SAEM (Monolix 2020R1)	IR (cytokines, granzyme B)	Parameters for base model adopted from [38]
Yang et al.^49^	Antiviral	No	Yes	^109,111,112^	4	38	SAEM (Monolix 2019R2)	VL, IR (antibodies)	
Phan et al.^74^	Antiviral	No (but taken into account)	Yes	^113,114^	9	2	NLME (Monolix 2021)	VL*	*With bamlanivimab infusion
Byrne et al.^68^	Antiviral	No (but possible problems mentioned)	Yes	^115^	9	160	Nelder-Mead via POMP package (R)	VL, IR (antibodies, CD4^+^ and CD8^+^ T cells, IFN-stimulated genes (ISGs)); all non-human	Not all models included in publication
Iyaniwura et al.^41^	Antiviral	Mentioned, not shown	No	^116^	5	7	NLME (Monolix 2021R1)	VL*	*Total viral RNA and infectious virus per patient
Ke et al.^35^	Antiviral	No	Yes	^35^	4, 5, 6	114*	NLME (Monolix 2019R2)	VL	Based on 5 base models with different fixed parameters
Schuh et al.^84^	Antiviral	Yes	Yes	^84^	4	2	fmincon() (MATLAB)	VL	
Fan et al.^48^	Antiviral	No	Yes	^86^	3, 4, 5, 6, 7	5	n.a.	VL	Many IR parameters from literature
Tan et al.^33^	Antiviral	No	Yes	^33^	4	4	Markov chain Monte Carlo (MCMC) (RStan)	VL	
Zitzmann et al.^28^	Antiviral	Yes	Yes	^88^	4, 5	2	SAEM (Monolix 2023R1)	VL	
Vaidya et al.^61^	Antiviral	Yes	Yes	^117^	4, 5, 5	3	ode45 (MATLAB 2020a)	VL (non-human)*	*Variants F13-E and CTan-H
Schuh et al.^39^	Antiviral	Yes	Yes	^35^	4	2	MATLAB 2023a	VL	
Ghostine et al.^50^	Antiviral	No	No	^86^	9	1	Ensemble Kalman filter (EnKF)	VL	Main focus on EnKF; IR parameters from literature
Gonçalves et al.^66^	Antiviral	No (but taken into account)	Yes	^118^	10, 11	2	SAEM algorithm (Monolix 2018R2)	VL (non-human)	IR as extension
Raach et al.^24^	Antiviral	No (but taken into account)	Yes	^24^	10, 11	2	Approximate Bayesian Computation (pyABC)	VL (in vitro)	IR as extension
Zhao et al.^73^	Coinfection	Yes	Yes	^119,120*^	9, 7, 7, 7, 7	5	Goodman-Weare Markov chain Monte Carlo (GWMCMC) algorithm	VL (HIV, SARS-COV-2), IR (CD4^+^ T cells)	*Preprint; HIV + SARS-CoV-2
Pinky et al.^75^	Coinfection	No	No	^93^	7	1	Nelder-Mead (Octave)	VL	Used only 1 patient
Pinky et al.^60^	Coinfection	No	Yes	^86,121*^^,^^122*^	4, 7, 8	42	NLME + SAEM algorithm (Monolix 2019R1); scipy.optimize.minimize (Python)	VL (non-human, coinfection); VL (human single infection)	*Viral load data, not analyzed here
Alexandre et al.^67^	Compartment	Yes	Yes	^123–125^	12	8 + 12	SAEM algorithm (Monolix 2019R1)	VL (non-human)	
Mochan et al.^65^	Compartment	No	Yes	^91^	9	2	fminsearchbnd() (MATLAB)	VL (non-human)	
Ciupe et al.^25^	Compartment	Yes	Yes	^86^	6	1	Delayed rejection adaptive Metropolis (DRAM) algorithm (MATLAB)	VL	
Dogra et al.^27^	Compartment	No	Yes	^126,127^	40	2	Least-squares (MATLAB)	VL (non-human, clinical), IR (antibodies, cytokines, lymphocytes)	Many IR parameters from literature
Goyal et al.^62^	Compartment	Yes (mentioned, not shown)	Yes	^91,128^	7 + 9*	55	SAEM + MCMC (Monolix)	VL (non-human)	*Different models
Chhetri et al.^83^	Simple	No	Yes	^86,129^	3	1	Mean squared error (MSE) (MATLAB)	VL, IR (cytokines)*	Fit not shown; no separate equations for immune response; *single timepoints
Lingas et al.^80^	Simple	No	Yes	^130^	5	2	SAEM algorithm (Monolix 2020R1)	VL	
Rodriguez et al.^63^	Simple	No	No	^131^	4	1	Sum of squared residuals (SSR) from scipy.optimize.minimize (Python)	VL (non-human)	
Korosec et al.^29^	Simple	Yes	Yes	^38,86,89,93,94,132^	4, 6	2	SAEM algorithm (Monolix 2020R1)	VL	
Dobrovolny^59^	Simple	No (but problems mentioned)	No	^128^	3, 1*	2	SSR with Nelder-Mead (Octave)	VL (non-human)	*Not a TCL model
Lingas et al.^56^	Simple	Yes	Yes	^56^	5	5	SAEM algorithm	VL, IR (antibodies)	No separate equations for immune response
Bernhauerova et al.^34^	Simple	No	No	^34^	n; >5	n*	Bayesian inference with Goodman and Weare affine invariant ensemble MCMC sampler (MATLAB)	VL (in vitro)	*n latent phases
Abuin et al.^76^	Simple	No	Yes	^86^	3	1	Differential Evolution algorithm	VL	
Patel et al.^79^	Simple	No (but taken into account)	Yes	^86,93,94,133–140^	4	1	SAEM	VL	
Kim et al.^31^	Simple	No	No	^86,93,136,141–144^	2, 3	5	SAEM (Monolix 2019R2)	VL*	*SARS-CoV-2, MERS-CoV, SARS-CoV
Torii et al.^64^	Simple	No	No	^64,145–147^	4	1	MCMC	VL (in vitro)	
McCormack et al.^32^	Simple	No (but taken into account)	Yes	^32^	5, 7	2	MCMC	VL* (in vitro)	*Omicron and Delta
Owens et al.^42^	Simple	No (but taken into account)	Yes	^88^	3, 4, 5	9	NLME	VL	Model includes early and late immune response
Odaka et al.^40^	Simple	No	Yes	^105^	3	4	Dynamic time warping	VL	Includes 3 models from other authors^148–150^.

## Overview of included studies

Viral dynamics are often explained using TCL models. The earliest known TCL models, developed in the 1990s, were described by refs. ^19–22^. A more recent paper by Baccam et al. presents two distinct TCL models for the influenza virus and is frequently cited in the studies reviewed here^23^.

**Fig. 2:**
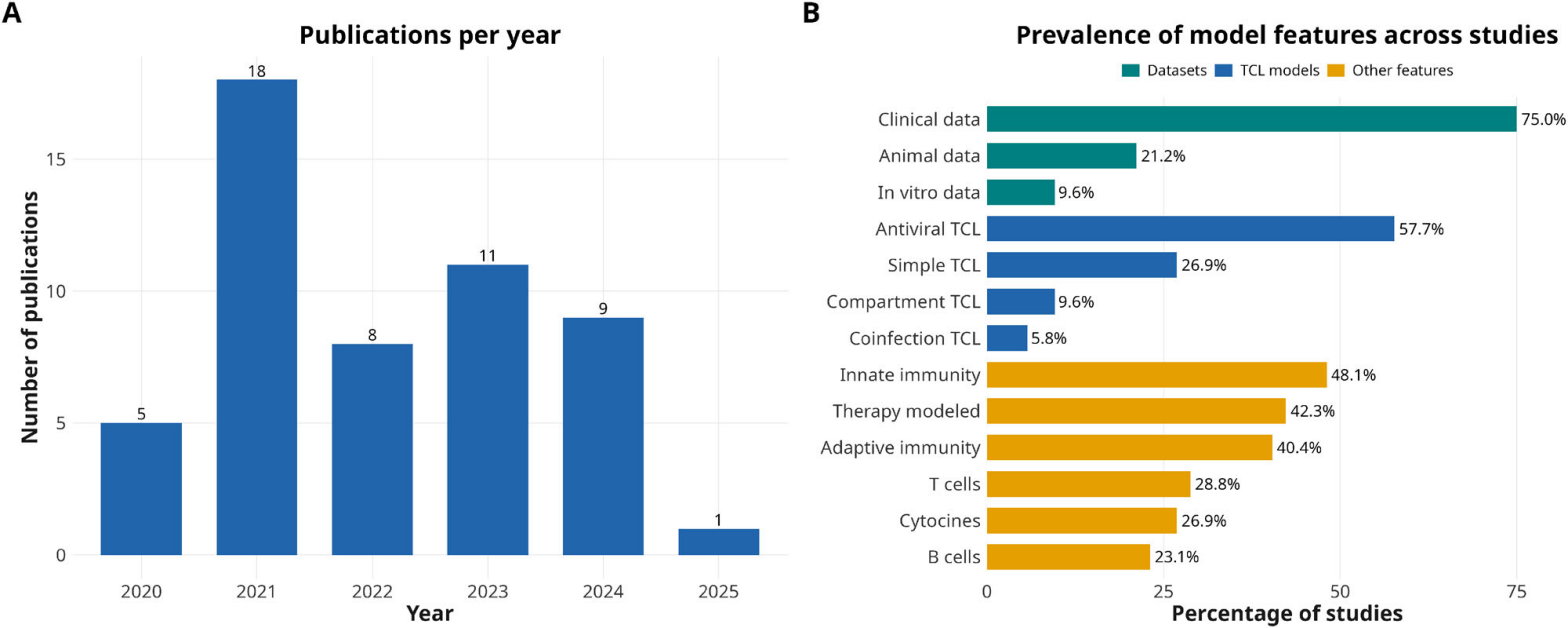
Trends and thematic coverage of modeling studies. **A** Number of peer-reviewed publications per year employing ODE models to study SARS-CoV-2 infections. The distribution highlights a sharp increase in modeling activity during the early phase of the COVID-19 pandemic (2021), followed by sustained but fluctuating output in subsequent years. This trend reflects the rapid adoption of quantitative viral dynamics models in response to urgent public health needs, as well as continued interest in refining model structure and data integration. **B** Percentage of included studies. Most models were calibrated using clinical viral load data, while fewer studies incorporated animal or in vitro datasets. Although extensions beyond simple TCL frameworks, such as antiviral treatment modeling and immune responses, were common, relatively few studies included compartmental structures or coinfection dynamics, highlighting gaps in model complexity and data availability.

In our review, we categorized all TCL models into the following categories based on their equations and complexity: simple TCL models, antiviral TCL models, coinfection TCL models, and (multi-) compartment TCL models (Fig. 2). Simple TCL models do not include explicit antiviral or immune-related state variables, operate within a single reaction compartment, and contain only one viral species. Implicit representations of immune responses or drug effects within the core equations are permitted. We identified 14 simple TCL models in our dataset. Their complexity ranges from 2 to 7 equations. Several models included in this review fall into this category. The most frequent category, however, consists of antiviral TCL models, comprising 30 studies. In this review, we classified models as antiviral TCL models if they include at least one state variable that explicitly represents an antiviral component, in contrast to simple TCL models in which such effects may be incorporated implicitly. Their complexity ranges from 3 to 32 equations. The third category includes (multi-) compartment TCL models. Here, we define compartments as biological, closed reaction spaces in which variables such as RNAs, viruses, proteins, or antibodies can enter or exit only through defined import or export processes. We identified five studies in this category, with model complexity ranging up to 40 equations. The final category comprises coinfection TCL models (*n* = 3), which must include at least two distinct viral species. These may represent different strains of the same virus or entirely different viruses. Their complexity reaches up to 9 equations. For more detailed information on the models, see Table 1 and Fig. S1. A detailed overview of all datasets used for the model fitting is summarized in Table 2 and Fig. S2.

**Table 2:** Overview of the datasets used for fitting the reviewed ODE models

Reference data	Study/measurement type	What was measured	Patients/ animals (*n*)	Data points per patient/replicate	Models using data	Comments
Ader et al.^130^	Randomized, controlled clinical trial	Viral load	832	Multiple timepoints per participant	^80^	
Agostini et al.^145^	In vitro study in human and animal cells	Viral load	n.a.	Multiple timepoints post-infection	^64^	
Al-Abdely et al.^144^	Clinical observational study	Viral RNA, antibodies, clinical signs	33	Multiple timepoints per participant	^31^	MERS study
Bernhauerova et al.^34^	In vitro study in human and animal cells	Viral replication kinetics	n.a.	Multiple timepoints post-infection	^34^	A549-ACE2 & Vero-E6 Cells
Böhmer et al.^90^	Clinical observational study	Viral load	16	Single-timepoint measurements	^26^	
Brouwer et al.^123^	Animal study	Viral load, antibodies, immune cells	Ten NHPs, five rabbits, eight mice	Multiple timepoints per animal	^67^	Cynomolgus macaques, mice, rabbits, patient sera, cells
Bryan et al.^111^	Clinical observational study	Viral load, IgG, WBC, lymphocytes, neutrophils, CRP	245	Single (and multiple in some cases) timepoints per participant	^49^	
Cervia et al.^112^	Clinical observational study	Viral load, antibodies	173	Multiple timepoints per participant	^49^	
Chan et al.^126^	Animal study	Viral load, antibodies, body weight, histopathological analysis	11/8	Multiple timepoints per animal	^27^	Viral load data for all organs (Syrian hamsters)
Cheng et al.^137^	Clinical observational study	Viral load, clinical signs	5	Multiple timepoints per participant	^79^	
Choudhary et al. and Chew et al., respectively^113,114^	Double-blind, randomized controlled phase 2 clinical trial	Viral load, clinical signs	317	Multiple longitudinal measurements	^74^	
Collier et al.^96^	Prospective observational study	Antibodies	78	Multiple timepoints per participant	^57^	
Corbett et al.^124^	Animal study	Viral load, antibodies, inflammatory markers	24 NHPs	Multiple timepoints post-infection	^67^	Appendix 2; Indian-origin rhesus macaques
Del Valle et al.^106^	Clinical observational study	Cytokines	1484	Single (and multiple in some cases) timepoints per participant	^53^	
Lingas et al.^56^	Clinical observational study	Viral load, antibodies	57	Multiple timepoints per participant	^56^	
Garzon et al.^103^	Randomized, controlled clinical trial	Pharmacokinetics data	95	Multiple timepoints per participant	^77^	
Han et al.^104^	Clinical observational study	Viral load*, immune cells	154	Multiple timepoints per participant	^82^	*No time series data
Han et al.^140^	Clinical observational study	Viral load	12	Multiple timepoints per participant	^79^	Children
Jones et al.^132^	Large-scale observational analysis	Viral load	25381	Typically, one RT-PCR (first positive), with a subset (4344 individuals) having ≥3 longitudinal tests	^29^	Some viral load data before viral peak
Karim et al.^119^	Longitudinal observational cohort study	Viral load, immune cells	236	Multiple timepoints per participant	^73^	In total, 55 different parameters
Karim et al.^120^	Case report	Viral load, antibodies, immune cells	1	Multiple timepoints	^73^	Preprint
Kawasuji et al.^138^	Clinical observational study	Viral load	28	Multiple timepoints per participant	^79^	
Ke et al.^35^	Prospective cohort study	Viral load	60	Multiple timepoints per participant	^35,39^	
Killingley et al.^116^	Human challenge study	Viral load, clinical signs	34	Multiple timepoints per participant	^41^	Complete infection Dynamic
Kim et al.^133^	Clinical observational study	Viral load	13	Multiple timepoints per participant	^79^	
Kim et al.^141^	Clinical observational study	Viral load, inflammatory markers, immune cells, clinical signs	28	Multiple timepoints per participant	^31^	
Kim et al.^89^	Case report	Viral load	2	Multiple timepoints per participant	^26,29,46,77,79^	
Kissler et al.^88^	Clinical observational study	Viral load	68	Multiple timepoints per participant	^28,42,44^	Viral load data before viral peak
Lescure et al.^94^	Clinical observational study	Viral load, antibodies, clinical signs	5	Multiple timepoints per participant	^29,46,51,77,79^	
Lin et al.^45^	Clinical observational study	Cytokines	23 (+healthy control donors)	Multiple timepoints per participant	^45^	
Long et al.^109^	Clinical observational study	Antibodies	285	Multiple timepoints per participant	^47,49^	
Lucas et al.^97^	Longitudinal prospective cohort study	Viral load, antibodies, immune cells	113	Multiple timepoints per participant	^57^	
Lui et al.^134^	Clinical observational study	Viral load	11	Multiple timepoints per participant	^79^	
Maisonnasse et al.^118^	Animal study	Viral load, inflammatory markers	31 NHPs	Multiple timepoints per animal	^66^	Cynomolgus macaques treated with hydroxychloroquine
Marlin et al.^125^	Animal study	Viral load, antibodies, inflammatory markers, immune cells	18	Multiple timepoints post-infection	^67^	Cynomolgus macaques; mice
Martinot et al.^146^	Case report	Viral load	1	Multiple timepoints	^64^	
McCormack et al. and Peacock et al., respectively^32,151^	In vitro study in human and animal cells, animal study	Viral load, immune escape	24 hamsters	Multiple timepoints post-infection	^32^	Peacock et al. preprint, same data 293T; Caco-3; Caco-2; Vero E6; BHK-21; hNECs; hamsters
Mukae et al.^100^	Double-blind, randomized controlled phase 2b clinical trial	Viral load, clinical signs	341	Multiple timepoints per participant	^54^	Ensitrelvir treatment
Mukae et al.^99^	Double-blind, randomized controlled phase 2a clinical trial	Viral load	69	Multiple timepoints per participant	^54^	Ensitrelvir treatment
Munster et al.^91^	Animal study	Viral load, histopathological analysis of lung tissue	8 NHPs	Daily swabs, necropsy at 7 days post-inoculation	^26,62,65^	
Néant et al.^38^	Clinical observational study	Viral load, (mortality outcomes)	665	Longitudinal viral load measurements (serial samples for 284 patients); mortality tracked for all	^29,38,46^	
Nelson et al.^115^	Animal study	Viral load, antibodies, immune cells, inflammatory markers, histopathological analysis	6	Multiple timepoints post-infection	^68^	Rhesus macaques
Oh et al.^142^	Clinical observational study	Viral load	17	Multiple timepoints per participant	^31^	MERS study
Padoan et al.^108^	Clinical observational study	Antibodies	37	Multiple timepoints per participant	^51^	
Pan et al.^85^	Clinical observational study	Viral load	82	Multiple timepoints per participant	^26,78^	
Peeters et al.^98^	Prospective longitudinal, interventional multicohort trial	Antibodies	200	Multiple timepoints per participant	^57^	Data were available upon request, cancer patients
Peiris et al.^143^	Clinical observational study	Viral load	75	Multiple timepoints per participant	^31^	SARS-CoV study
Qin et al.^129^	Clinical observational study	Lymphocyte counts & subsets, leukocytes, NLR, monocyte/eosinophil/basophil percentages; inflammatory biomarkers and cytokines	452	Single-timepoint measurements	^83^	No time series data
Raach et al.^24^	In vitro study in human cells	Viral replication kinetics	n.a.	Multiple timepoints post-infection	^24^	Ciliated, basal, and secretory cells
Rockx et al.^131^	Animal study	Viral load*, histopathological analysis of lung tissue	12 NHPs	Daily swabs, necropsy at 7 days post-inoculation	^63^	*Viral load for MERS-CoV & SARS-CoV 2
Schuh et al.^84^	Prospective cohort study	Viral load	369	Multiple timepoints per participant	^84^	
Shi et al.^117^	Animal study	Viral load per organ, antibodies	Eight ducks, eight chickens, eight pigs, seven dogs, six ferrets, seven cats	Multiple timepoints per animal	^61^	Ferrets, cats, dogs, pigs, chickens, ducks
Singanayagam et al.^107^	Clinical observational study	Viral load	163	Multiple timepoints per participant	^36^	
Szemiel et al.^147^	In vitro study in human and animal cells	Viral load, escape mutations	n.a.	Multiple timepoints post-infection	^64^	
Tan et al.^33^	National swab-and-send-home database (Singapore Ministry of Health)	Viral load	159178	Multiple timepoints per participant	^33^	Access with permission, many different variants
Thevarajan et al.^135^	Case report	Viral load, immune cells	1	Multiple timepoints	^79^	
To et al.^87^	Clinical observational study	Viral load, antibodies	23	Multiple timepoints per participant	^43,47^	
Torii et al.^64^	In vitro study in human and animal cells	Viral replication kinetics	n.a.	Multiple timepoints post-infection	^64^	Syrian Hamster, HEK293-C34 cells
Vetter et al.^127^	Clinical observational study	Viral load, immune cells	5	Multiple timepoints per participant	^27^	
Warren et al.^102^	Animal study	Pharmacokinetics data	3	Multiple timepoints per animal	^77^	
Williamson et al.^128^	Animal study	Viral load, histopathological analysis	12 NHPs	Daily swabs, necropsy at 7 days post-inoculation	^59,62^	Rhesus macaques
Wölfel et al.^86^	Clinical observational study	Viral load, infectious virus isolation, sgRNA, viral sequencing, antibody seroconversion	9	Multiple timepoints per participant	^25,29,31,44,46,48,50,55,60,76–79,83^	
Yazdanpanah et al.^92^	Clinical multicentric prospective cohort study	Viral load	246	Multiple timepoints per participant	^38^	Subcohort from^38^
Yoon et al.^139^	Case report	Viral load	2	Multiple timepoints	^79^	
Young et al.^93^	Clinical observational study	Viral load, antibodies, clinical signs	18	Multiple timepoints per participant	^29,31,46,75,77,79^	
Zheng et al.^105^	Retrospective cohort study	Viral load, antibodies	96	Multiple timepoints per participant	^40,82^	
Zhou et al.^110^	Retrospective study	Viral load, inflammatory markers, immune cells, clinical signs	191	Multiple timepoints per participant	^47^	
Zhou et al.^46^	Clinical observational study	Viral load, immune cells, cytokines, antibodies	213	Multiple timepoints per participant	^46^	
Zhou et al.^95^	Clinical observational study	Viral load, antibodies, immune cells	41	Multiple timepoints per participant	^46^	
Zou et al.^136^	Clinical observational study	Viral load	18	Multiple timepoints per participant	^31,79^	

We also examined whether the studies included sensitivity or identifiability analyses, both of which are important for the interpretability and reliability of the inferred model parameters (Table 1 and Fig. S3). Most models reported a sensitivity analysis, but only a few performed an identifiability analysis. While formal identifiability analysis may not be required for models aimed at prediction or comparison rather than mechanistic interpretation, its absence can limit the interpretability and comparability of parameter estimates across studies. Sensitivity analyses are generally necessary for TCL ODE models, as their nonlinear dynamics can be strongly affected by small parameter changes—meaning that good model fits alone do not ensure robust conclusions.

## Summary of literature

### Virus replication dynamics

In this chapter, we examine general viral replication dynamics within the human body without addressing immune response or treatment dynamics. Conceptually, virus replication models focus on three different scales: cell level, tissue level, and population level.

For the tissue scale level, within-host models of SARS-CoV-2 focus on the respiratory tract and target cells expressing ACE2^4,24^. Main target sites are the lower respiratory tract (LRT), upper respiratory tract (URT), and nasopharyngeal cells in the nose^25,26^. Only one study simulates infections in extrapulmonary organs^27^. For example, Raach et al. developed a tissue-dependent simple TCL model that models various cell populations in the respiratory tract and characterizes different infection dynamics there, leading to the conclusion that ciliated epithelial cells are the main target of SARS-CoV-2 infection based on the concentration of ACE2 receptors^24^. These tissue-based models indicate how anatomical structures are able to influence parameter estimation and viral kinetics, revealing that whole body dynamics cannot be reliably inferred from single tissue models.

To reliably estimate early virus dynamics within patients, several studies have investigated whether the data basis for such estimates is sufficient. They identified that an absence of data prior to the viral peak can lead to unidentifiable model parameters, which in turn lead to unreliable predictions for optimal treatment windows and may lead to underestimation of the period of peak infectiousness^25,28,29^. We examined the available data (see Table 2) and found this problem in almost all patient-based datasets. To address the challenge of missing early-stage clinical data, in vitro experiments offer a valuable alternative. Several model studies used in vitro datasets to compare different replication dynamics of MERS-CoV, SARS-CoV, and SARS-CoV-2 variants within different cell lines like A549-ACE2, Calu-3, Vero-E6, and nasopharynx cells, which show different characteristics and replication dynamics depending on the virus variant^30–34^. For instance, the SARS-CoV-2 Delta variant shows an advantage over the Omicron variant in Calu-3 cells, while Omicron shows a fitness advantage in human nasal epithelial cells, likely due to TMPRSS2-dependent pathways^32^. These studies illustrate an important issue within the field: variant-specific model calibration is essential, as fitness differences can shift depending on viral variant and experimentally chosen cell lines. Finally, population level variability has emerged as a major theme, as viral load and infectivity vary widely between individuals^23,27,33,35–40^. Iyaniwura et al. proposed a nonlinear relationship between the total viral RNA and infectious virus^41^. One of the most comprehensive studies to date, involving a cohort of 2875 individuals, was conducted by Owens et al., who systematically characterized the heterogeneity of SARS-CoV-2 viral kinetics in humans^42^. Through clustering analyses, six distinct patterns of viral progression were identified, according to differences in peak viral load, duration of infection, and clearance dynamics: low peak + early clearance, early clearance, late peak, high peak, late and low peak + late clearance and high peak + late clearance. Owens et al. predicts that these different patterns may be explainable by differences in timing and intensity of immune response. These patterns offer potential utility for diagnostic stratification and individualized monitoring^42^.

These studies highlight that model predictions are strongly influenced by the choice of cell types, viral variants, clinical datasets, and the temporal resolution of the data. Datasets lacking early timepoints should ideally be avoided if early predictions are required, or greater efforts should be made to generate data that capture these critical early stages. Careful selection of cell lines and viral variants is essential, as both can substantially affect experimental results. Finally, adjusted measurement strategies should be considered in preparation for future pandemics.

### Immune response

The host immune response is generally represented through interactions between the innate and adaptive immune components. In this framework, the innate immune response was modeled as a rapid, non-specific response mediated by macrophages and natural killer (NK) cells, whereas the adaptive immune response was assumed to act more slowly but specifically, via antibodies and plasma cells or T cell-mediated effects.

#### Innate immune response

A small subset of three immune response models focused exclusively on innate immune response, aiming to capture the complex interplay between viral replication and innate immune activity, providing insight into early viral control mechanisms prior to adaptive immune response.

Leander et al. focus on the innate immune response in the alveolar epithelium. Their model predicts that the initial viral load does not affect the peak viral load, but the number of ACE2^+^ cells significantly influences disease progression; consequently, increased spike protein-ACE2 binding of SARS-CoV-2 variants’ affinities may cause more severe outcomes. The study further predicted that interferon (IFN) treatment should be administered early, in small doses at short intervals, and combined with other treatments for optimal results. Simulations also indicate that delayed or excessive IFN signals can worsen prognosis. However, IFN therapy alone is not sufficient; they predict that only treatments with at least 90% efficacy can effectively control the infection^43^.

Wang et al. predicted similar results, which also include early IFN treatment in combination with antiviral drugs. The model also indicates lymphocytes as a secondary target of SARS-CoV-2 infection, potentially contributing to delayed or dysfunctional immune responses^26^. Further predictions suggested substantially stronger immune activity and viral production in the LRT compared to the URT. Furthermore, Ke et al. proposed that the logarithm of viral load in the URT, rather than the viral load itself, serves as a more accurate indicator of infectiousness due to a nonlinear relationship between viral load and transmission potential^44^.

Collectively, these studies highlight the central role of the innate immune response in the early control of SARS-CoV-2. However, the small number of studies exclusively analyzing innate immune response limits generalization. Across the models, the innate immune response was insufficient to clear infection, highlighting early timing of treatment and dosage of immunomodulatory interventions as critical key factors against infection progression.

#### Adaptive immune response

Across included studies, the adaptive immune response was mainly modeled via T cell-mediated cytotoxicity and antibody dynamics. CD4^+^ T cells were modeled as coordinators of cytokine production and immune activation, while CD8^+^ T cells directly contribute to the elimination of infected cells. B-cell-derived antibodies, such as IgG or IgM, were included as neutralizing components, with some models explicitly accounting for delayed antibody responses and memory functions.

Several studies emphasized a prominent role of T cells for controlling the SARS-CoV-2 infection, disease severity, and long-term protection, although the magnitude of this effect varied across model structures and parametrizations^45–47^.

Furthermore, cytotoxic T cell activity was identified to be more influential for viral clearance than natural killer cell-induced effects, particularly in the lung compartment^48^. Antibody-induced neutralization was predicted to have a limited impact during early infection due to delayed antibody production relative to the viral peak^49^; however, early adaptive immune activation correlates with a milder course of disease^46,50^. Antibody dynamics were explored heterogeneously across models, including representations of non-neutralizing responses and antibody-dependent enhancement (ADE)^51^. These simulations demonstrated that high antibody concentrations without sufficient neutralizing capacity could exacerbate target cell destruction and fail to achieve viral clearance. As a result, antibody responses were identified as potentially protective or detrimental, depending on their timing, concentration, and neutralization efficacy^46,51,52^. Lin et al. observed that severe disease progression is associated with increased IL-2 production and reduced granzyme B levels^45^. This appears paradoxical, as IL-2 promotes the production of CD8^+^ T cells, which in turn produce granzyme B. The hypothesis suggests that this phenomenon may be due to a functional dysregulation induced by SARS-CoV-2, resulting in decreased granzyme B concentrations. A possible explanation by Reis et al. could be that dysfunctions can arise from the potential infection of macrophages, CD4^+^ T cells, and CD8^+^ T cells, which may ultimately lead to a cytokine storm^47^.

The cytokine storm, also known as cytokine release syndrome (CRS) or hypercytokinemia, as a consequence of adaptive immune dysregulation, was investigated via two models. Predictions suggest that an infection of antigen-presenting cells and lymphocyte populations could lead to increased production of several pro-inflammatory cytokines, primarily IL-6, leading to widespread damage of healthy tissue^47^. Simulations incorporating immunomodulatory therapy suggest treatment with cytokine inhibitors had the most beneficial effect on protecting healthy cells and tissue^53^.

These antiviral models identified adaptive immunity as an important protective or pathogenic factor for SARS-CoV-2 disease progression. Early, coordinated adaptive responses favor viral clearance, whereas delayed or dysfunctional responses promote hyperinflammation, immune dysregulation, and severe outcomes. Memory cells and IFNs appear central to both short- and long-term control of the virus, underscoring the adaptive immune response as a key determinant of viral clearance and durable protection.

### Vaccination

Across antiviral TCL models, vaccination was incorporated as a modifier of host immunity and/or viral kinetics. In these models, vaccination was associated with a significant reduction in disease severity due to a more robust immune response^28,44,54^ or due to significantly shorter duration of high viral load^55^. Predictions further indicated that individuals with high antibody titers following infection or vaccination, who experienced mild to moderate symptoms^56^, or who remained asymptomatic when re-exposed^49^, do so due to strong immune response mechanisms. Within the included studies, Dogra et al. developed a COVID-19 vaccination plan for general vaccine-induced protection based on clinical data. They differentiated between immunocompetent and immunocompromised individuals and tailored vaccination plans to meet the specific needs of each group. Their model identified differences in CD4^+^ T cell activation between these groups, recommending that immunocompromised individuals receive booster doses at shorter intervals to ensure optimal protection^57^. Comparative modeling of vaccine types indicated differential effects on viral dynamics depending on the vaccine platform and circulating variant. Tan et al. reported that mRNA vaccines (Pfizer, Moderna) were more effective than non-mRNA vaccines (Sinovac, Sinopharm), which was particularly evident in delta infections. With omicron, the difference was not as pronounced^33^.

Across all included antiviral TCL models that compare vaccination with drug therapy or immune responses, vaccination is consistently concluded to provide the best protection against COVID-19 within the respective model assumptions and parameter ranges.

### Animal models

Animal testing has long been an essential component of disease research and treatment development, as certain animal species exhibit significant similarities to humans in various aspects of infection and disease progression^58^. Animal models have also been used to study SARS-CoV-2 infection dynamics, as well as vaccination strategies and antiviral treatment. Most of the studies focus on rhesus macaques (see Table 2), but there are also studies available from ferrets, hamsters, and mice^27,59–61^. The generated datasets substantially differ from clinical datasets due to complete infection dynamics, especially for the early timepoints.

Several models were used to investigate the drug treatment with remdesivir in macaques^59,62,63^ and hamsters^64^. Model predictions suggest that the drug efficacy can be influenced by host age (young vs. old), viral mutations (within NSP12), and timing of drug administration (before, after viral peak), leading to prolonged survival of infected cells^59,63,64^. For instance, Goyal et al. used a compartmental model to predict that remdesivir treatment leads to an increased viral load in the nasal passages due to the absence of refractory cells^62^. Thus, they suggest that nasal swab data may not serve as a reliable indicator of lung disease in the context of partially effective antiviral treatments such as remdesivir. Other models also indicate that due to vaccination, prophylactic and early treatment, tissue damage can be reduced and therefore severe illnesses can be prevented^65–67^.

Immune-response modeling highlights that LRT infection relies mainly on innate and inflammatory mechanisms, whereas URT clearance depends on adaptive immunity^65^. In general, key mediators, such as IFI27 and CD4⁺ T cells, are responsible for viral clearance^68^—and additional models in ferrets demonstrate that infection order in coinfections can alter disease outcomes^60^, as previously demonstrated for influenza^69^.

Rhesus macaques and other animal models show valuable mechanistic insights into SARS-CoV-2 infection dynamics, immune responses, antiviral treatments, and vaccination. They allow measurements of early infection dynamics that are often missing in clinical datasets. They show that viral clearance depends on host factors, treatment windows, and the underlying immune response, although species-specific differences can limit direct comparisons to humans.

### Coinfection

Epidemiological observations indicate that coinfections occur in a substantial fraction of patients^70,71^, but only one of the included studies was based on clinical coinfection data between human immunodeficiency virus (HIV) and SARS-CoV-2 in patients from South Africa^72^. All remaining coinfection modeling studies are based on animal or in vitro experiments (Table 2).

Model results from real clinical data suggested that antiretroviral therapy alone did not substantially reduce SARS-CoV-2 viral loads^72^. Further simulations with animal or in vitro data indicated that combination strategies targeting both HIV and SARS-CoV-2 were associated with improved CD4^+^ T cell levels and reduced SARS-CoV-2 particle counts, whereas the absence of antiretroviral therapy led to pronounced CD4^+^ T cell depletion^73^. SARS-CoV-2 also may reactivate latent HIV reservoirs, leading to an increased HIV viral load^73^.

Another study explores viral rebound following bamlanivimab (BAM) administration, an anti-spike monoclonal antibody^74^. The model operates under the assumption that some viral strains exhibit resistance to BAM. It suggests that variations in the target cell supply rate and the adaptive immune response may contribute to the observed rebound. Specifically, the rapid reduction in infected cells due to BAM treatment leads to decreased IFN production. This, in turn, results in fewer target cells transitioning into a refractory state, thereby increasing their susceptibility to reinfection.

A small number of within-host models examined coinfection dynamics between SARS-CoV-2 and other respiratory viruses, primarily influenza A virus (IAV)^60,75^. Building on established influenza modeling^69^, these studies explicitly incorporated infection order and timing, and both studies conclude that the order of infection matters, which can lead to severe disease or death.

Simulations by Pinky et al. demonstrated that when a faster-replicating virus infects the host first, it can dominate early infection dynamics by inducing a strong innate immune response, particularly IFN production, thereby suppressing subsequent SARS-CoV-2 replication. In contrast, delayed introduction of the second virus favored SARS-CoV-2 dominance, while intermediate timings resulted in more balanced coinfection dynamics^60,75^.

In summary, the included models consistently identified infection order, timing, and immune interactions as key determinants of coinfection outcomes. But due to limited availability or non-existence of coinfection data, these conclusions should be interpreted with caution within the limits of the underlying model assumptions and data sources.

### Drug therapies

Across the included TCL ODE models, antiviral drug therapy was primarily investigated to identify optimal therapeutic windows, effective molecular targets, and possible outcomes for drug therapy.

Several modeling studies consistently identified the period prior to the viral load peak as the critical therapeutic window^76,77^. Antiviral drug administration was most effective when initiated within 3 to 5 days post-infection^31,78–81^, whereas monotherapy treatment after the viral peak was predicted to have little to no impact on viral dynamics^31,82^. However, early antiviral therapy has also been associated with subsequently delayed disease resolution^76,77,79^, potentially impairing the immune system’s capacity to clear the virus. Drug administration 7–10 days post-infection has virtually no influence on viral dynamics, as the majority of host cells are already infected by this stage^78–80^. Predictions suggest that post-peak treatment can only be successful if it involves combination therapy comprising two or more distinct drugs, preferably targeting different inhibitory mechanisms or incorporating immunomodulatory functions^31,76,79,82,83^.

A substantial number of antiviral models examined possible drug targets for combination and monotherapy approaches. Strong focus lies on replication inhibition drugs, like the nucleotide analog remdesivir^31,76–80,82–84^. Mathematical models consistently identify replication inhibition as a highly effective antiviral strategy, particularly when integrated into combination therapies or initiated early. The study by Schuh et al. reported limited clinical impact of remdesivir when treatment occurred late, suggesting that delayed intervention rather than insufficient drug efficacy explained poor outcomes within the model framework^84^. Alternative modeling approaches focused on inhibition of viral binding and cellular import mechanisms^31,36,76,79^. Simulations indicate that early administration of entry inhibitors could reduce viral load; however, comparative analyses suggest that suppression of viral replication/production yields a greater impact on reducing viral load compared to blockade of viral attachment/infection^55,82^. Goyal et al. conducted the only study that investigates export inhibition via selinexor^77^. Across all analyzed models, combination therapy was consistently predicted to outperform monotherapy. However, the magnitude of predicted benefit from combination therapy depends on explicit representation of synergistic mechanisms within the model structure and may be a trivial outcome of the modeling framework rather than a meaningful biological insight.

In general, simulations conclude that treatment timing and drug inhibition mechanism are important. Early interventions targeting viral replication with combination treatment were predicted to be most effective, while delayed treatments are followed by severe disease outcomes. Alternative drug targets besides viral import/export and viral replication remain unexplored.

## Conclusions

Modeling of SARS-CoV-2 expanded rapidly around 2020/2021 with the availability of initial clinical datasets and has continued at a steady pace since then. Most models focus on viral dynamics and treatment effects, often with only simplified representations of immune responses. However, most studies rely on retrospectively collected datasets rather than data specifically generated for modeling purposes, which introduces inherent limitations. In particular, the scarcity of comprehensive immune profiling data restricts the ability of current models to mechanistically capture immune responses.

Here, we present a comprehensive analysis of available TCL ODE models of within-host SARS-CoV-2 infection dynamics. The current state of computational TCL ODE models focuses mainly on replication dynamics, immune responses, and therapeutic strategies for SARS-CoV-2 infections. Across the included studies, it has been predicted that early drug intervention before viral peak or immune response favors the outcome. Delayed drug administration or late activation of the immune system led to severe disease progression or death. Combination therapy is always preferred over monotherapy by the models, ideally with different drug targets, but it may be a result of underlying model structures. The adaptive immune system is more important than the innate immune system, but it can also be part of severe disease progression if dysregulations occur. Drug intervention mainly focuses on import/export and replication, whereas replication seems to be the preferred drug target, but it neglects other potential drug targets.

It has been emphasized that the selection of cells/tissue and virus variants can strongly influence the results. Therefore, generalizable model predictions are not always possible and are also strongly influenced by the underlying assumptions, data and equations.

A major limitation across the data from human cohort studies used for the modeling studies is the scarcity of high-quality clinical data capturing early infection dynamics prior to the viral peak, leading to unidentifiable parameters and unreliable early predictions. Furthermore, there are likely time-series datasets that have not yet been utilized by any mathematical model and are therefore not included in this review. Another limitation is the focus on simple ODEs and TCL models, removing other modeling approaches and model structures.

While current models describe viral behavior across cell lines and patients, interactions with the immune system (particularly in long COVID and severe immune responses) remain less explored. Differences between variants are also rarely systematically addressed. Existing frameworks could support such analyses, offering a basis for better understanding disease dynamics and variant-specific patterns. Future modeling work should also incorporate new drug targets and more complete datasets in order to obtain a more comprehensive picture of SARS-CoV-2 dynamics and further improve the predictive power of mathematical models. Furthermore, the difference in replication dynamics between the SARS-CoV-2 variants is touched upon from time to time, but they have not yet been fully clarified for most of the variants and virus species.

## Supplementary information

Supplementary information.

## Data Availability

All data discussed in this review are available within the published articles cited in the manuscript, unless stated otherwise.
