# Supplementary material for: Ordinary differential equation models of SARS-CoV-2 replication dynamics and antiviral drug efficacies
Source: Npj Viruses. 2026 Mar 13;4:17. doi: 10.1038/s44298-026-00183-8 (PMC12988066; doi:10.1038/s44298-026-00183-8)
Supplement: Supplementary file 1 — Supplementary information. [file 44298_2026_183_MOESM1_ESM.pdf]

Supplementary information

Ordinary differential equation models of SARS-CoV-2 replication dynamics and antiviral drug efficacies

Kapischke et al., 2026

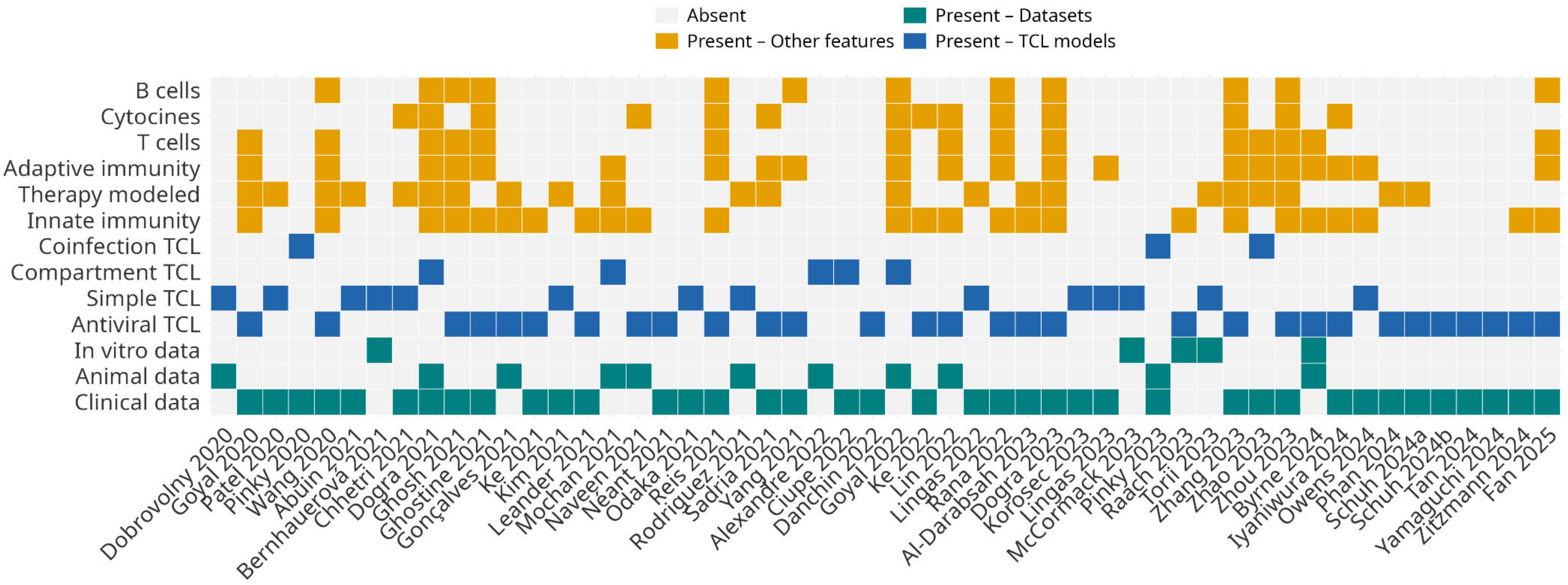

Figure S1. Overview of model features and data sources across included studies.

Map summarizing the presence or absence of key modeling components, biological processes, and data sources across all 52 included studies. Columns correspond to individual publications (ordered chronologically), and rows indicate whether specific features were included, such as simple or extended TCL models, antiviral treatment modeling, immune components (innate, adaptive, cytokines, T cells, B cells), coinfection or compartmental formulations, and the use of clinical, animal, or *in vitro* data. Colors indicate the presence of a given feature, while light grey shading indicates absence. The figure highlights substantial heterogeneity in model structure and data usage, as well as the limited overlap of multiple data types within single studies.

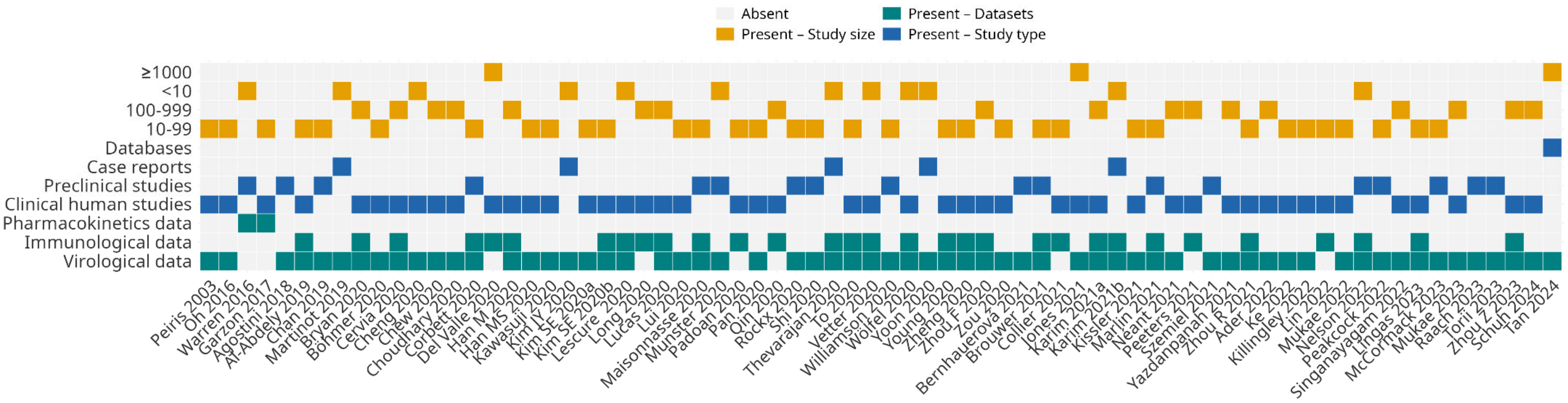

Figure S2. Overview of dataset features used in the reviewed models.

Map summarizing the availability and characteristics of data sources across all reviewed models. Rows indicate dataset features and study characteristics. Colored cells denote presence of a feature, while light gray indicates absence. This visualization highlights heterogeneity in data types and sample sizes used to inform the reviewed models.

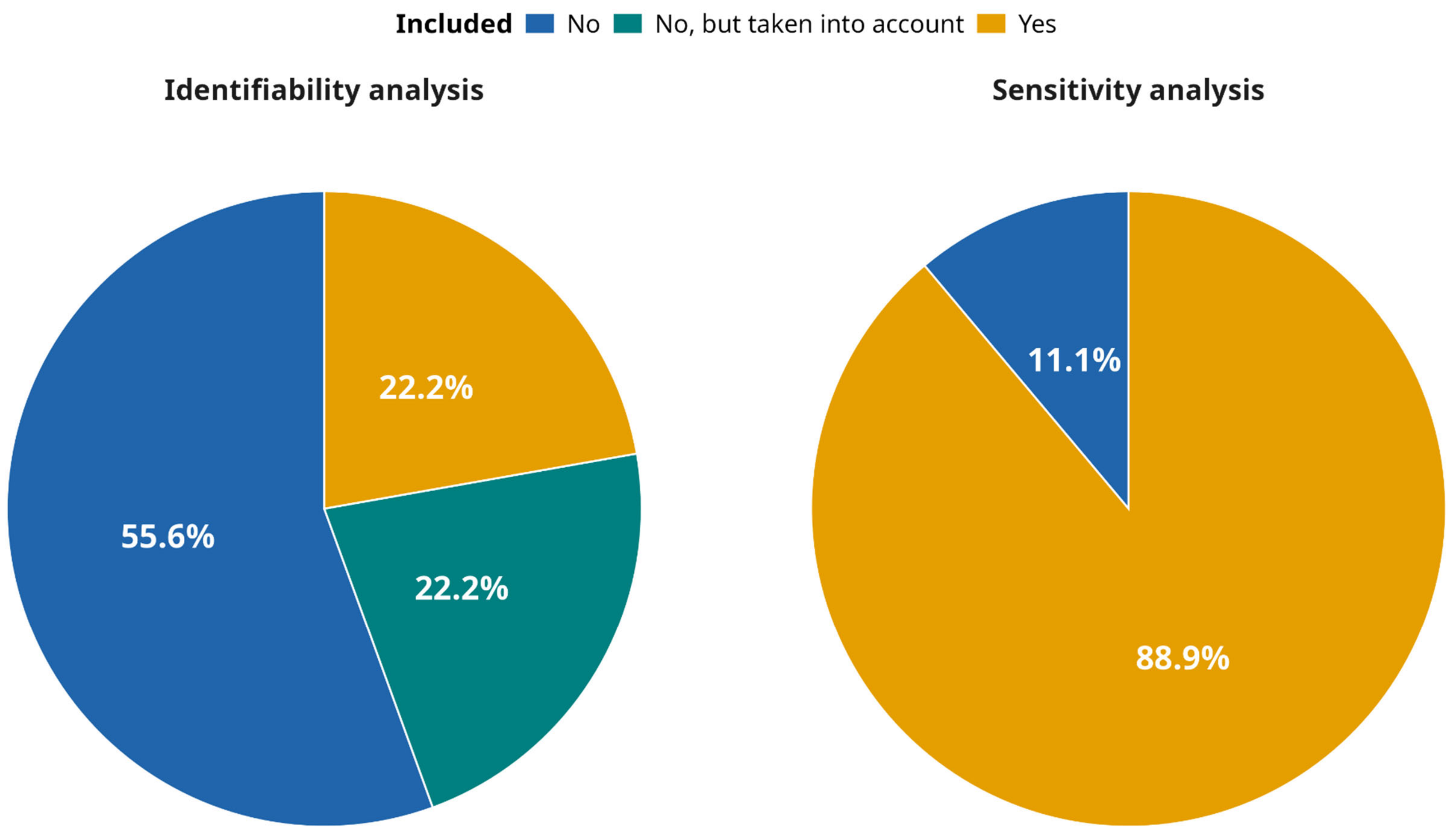

Figure S3. Inclusion of identifiability and sensitivity analyses in the reviewed studies.

Pie charts show the proportion of studies that reported identifiability (left) and sensitivity (right) analyses as included, not included but taken into account, or not included.
